# Research priorities in medical education at Shiraz University of Medical Sciences:categories and subcategories in the Iranian context

**Published:** 2016-01

**Authors:** PARISA NABEIEI, MITRA AMINI, SHIRIN GHANAVATI, SAADAT MARHAMATI

**Affiliations:** 1 Quality Improvement in Clinical Education Research Center, Education Development Center, Shiraz University of Medical Sciences, Shiraz, Iran;; 2 Shiraz University of Medical Sciences, Shiraz, Iran

**Keywords:** Research priorities, Medical education, Delphi method

## Abstract

**Introduction:**

Research in education is a globally significant issue without a long history. Due to the importance of the issue in Health System Development programs, this study intended to determine research priorities in medical education, considering their details and functions. By determining barriers existing in research in education progress, it is tried to make research priorities more functional by recommending acceptable strategies.

**Methods:**

This is a qualitative-descriptive study in two descriptive phases. The goal of these phases was to determine research priorities subcategories in medical education by Nominal Group Technique (NGT) and two rounds of Delphi method. Through the first phase, subcategories of research priorities were determined, using Nominal Group Technique under medical education experts’ supervision. Through two rounds of Delphi, a questionnaire was constructed based on the subcategories. Eventually, research priorities were determined based on their highest score (scores more than 7 out of 10).

**Results:**

In the first phase (NGT), 35 priorities in 5 major fields of medical education were presented. In the second phase, priorities were scored, using Delphi method. Medical Ethics and professionalism gained the highest scores (7.63±1.26) and educational evaluation the lowest (7.28±1.52). In this stage, 7 items were omitted but 2 of them were added again after experts’ revision in the third round of Delphi.

**Conclusion:**

According to the results of the present study and based on previous studies, it really seems that the fields of “Learning and Teaching Approaches” and “Medical Ethics and Professionalism” were more important. Because of financial and resource limitations in our country and the importance of research priorities, it is recommended to frequently study “research priorities determination program” at universities.

## Introduction

Research in Medical education was initiated in Buffalo University of New York about 4 decades ago in the form of a project. Afterwards, research in educational issues was considered as one of the major issues in medical education. Success and improvement in these issues are based on employing researchers, physicians and young professors who can train motivated, creative and knowledgeable students (1-3).

Determining research priorities is a process through which all potential research titles and proposals, regardless of their quality and priority, are determined. In other words, in the identification of research needs, achieving maximum of research titles and issues is the main goal which can be met through various research techniques such as Delphi and Expert panels (4-6).

Amini, et al. in the study conducted in fields of training physicians to be effective teachers; community-driven models for curriculum development; clinical teaching models; education on professionalism and ethics; and education for evidence-based medicine concluded that paying more attention to research priorities was necessary (7).

Tootoonchi in another research entitled “Research Priorities in Medical Education: A National Study” in 2012 found the following issues as the most important and major research priorities in Iran: Faculty Members Development Approaches, Providing motivation for both professors and students, Faculty members’ promotion policy, Learning Strategies, etc. (8).

Obeidat etal. in a study entitled “Reprioritizing current research trends in medical education: A reflection on research activities in Saudi Arabia” published in Medical Teacher Journal in 2015, came to the conclusion that the most important and major barriers in research development in Medical Education, were lack of proper research environment for researchers to conduct their activities, limited research budget, and lack of research at clinical environments (9).

As Shiraz University of Medical Sciences offers medical educationat two levels of Master of Science and PhD degrees, identifying research priorities for the students’ theses and dissertationsis of high significance (10-11). This not only helps the development of research in the country but also aids in enhancing the students’ thesis quality (12). In this connection, the purpose of the present study was to determine more detailed and applicable research priorities.

## Methods

This qualitative-descriptive study was conducted to determine subcategories of research priorities, using two-round Delphi method and NGT. The target population of the study consisted of medical education experts. According to this study as a qualitative one, purposeful sampling method was used to select experts, so that sample size was not predetermined and data gathering was continued till saturation was reached. Saturation occurs when there is no new information from experts (13-15). It is worth mentioning that the inclusion criteria of the study was based on the participants’ willingness to participate in the study, and the exclusion criteria was unwillingness of the participants to continue the study or not fulfilling the questionnaires through 2^nd^ and 3^rd^ rounds of Delphi method.

The NGT method was used to achieve the study goals. In this phase, the Nominal Group Technique was used to design the study properly. A group of elite consisting of 13 faculty members and general practitioners working at Education Development Center were invited to participate in an Expert Panel. The meeting began by introducing research priorities in medical education derived from literature (15) to experts and emphasizing that there was a need to justify sub-categories of research priorities in each field of medical education.

The participants were asked to think about sub-categories of each research priority and write their responses on a piece of paper privately. Then each participant was asked to name one sub-category of research priorities at a time and this idea was mentioned on the board by the leader. This process continued till all participants’ ideas were written down on the board, then each sub-category was discussed completely. Repeated ideas were omitted and integration of ideas was done.

Then the leader of the study asked each member to score each idea out of 10. Score 10 was the most important idea and score 1 was the least important one. Firstly, the leader asked the participants to score their ideas and then to categorize them from the most important one to the least important in their lists containing Medical Education Research Priorities tables. Then the researchers gathered the data and wrote them down on the board in order to calculate the mean score for each idea. In the next step, each question which was scored was written on the board for every participant to see. Scores of the questions were also written.

The researchers discussed the questions’ scores more in order to clarify everything for the participants and make them understand the meaning of each priority properly.

In the sixth step, each participant scored 5 important priorities on the board. The mean score was considered for these scores repeatedly. In the last step, the obtained scores were again discussed. The data gathered from the Nominal Group Technique were analyzed by SPSS software version 14 and the mean score of experts’ ideas was determined.

After Nominal Group Technique was conducted, 35 priorities were obtained as sub-categories of the major fields in Medical Education which were designed as a questionnaire. The validity of the mentioned questionnaire was achieved after determination of the variants and sub-categories in this phase. In order to do this, the opinions of 12 Medical Education experts were considered. In this section (face validity of the questionnaire), the questions were sort based on Medical Education major section accurately. Questions related to a specific field were categorized together. Firstly, general items and then specific items were mentioned. Sensitive or threatening items were placed at the last part of each item categorization. Then participants were asked to determine the weight of each item based on the questionnaire scale“1-5”. The last mentioned method was “impact scare” method (15). In order to check the reliability of the questionnaire, a pilot study was done among a group of participants of research workshops held at Education Development Center and the data derived from this section were assessed using Conbach’s Alpha.

**Second Phase: Delphi Model**

In order to conduct this section of the study which was simultaneous with 16^th^ congress of medical education held in Tehran, by purposeful sampling method some of the medical education experts of the country were selected to develop the priority questionnaire. In this step, scores1to10 were used to organize and categorize experts’ ideas.

In the second round of Delphi, Data gathered from the questionnaires were analyzed by SPSS software version 14 and their mean score and standard deviation were calculated. Priorities which achieved 70% of experts’ ideas were considered as the most important and most related subcategory based on experts’ ideas, and the ones which achieved less than 70% were omitted.

Based on the data gathered from previous sections, in this step, the participants could change or decrease their previous scores. They were also asked to explain the reason why they changed the scores which were achieved through previous sections. Finally, the questions that achieved higher scores by all experts participating in the study were considered as the major sub-categories of each field in Medical Education. It is worth mentioning the final list of major items in each field of Medical Education was e-mailed to the participants.

**Medical Ethics Considerations:**

This study as a qualitative study did not have any threats for the participants and its purpose was only to improve future planning of research proposals. This issue is announced in the introduction, questionnaire and at NGT meetings, so there was no need to take any informed consents from the participants.

It is also worth mentioning that, in the present study, there was no disclosure of the participants’ name or any other information; the participants were informed about results of the study or probable use of its results in any section of the study. 

## Results

In order to determine the sub-categories of research priorities in various fields of Medical Education, 13 experts of Medical Education Group participated; more than half of the mentioned group aged less than 50. 76 percent of the participants were male and 24 percent were female. At the end of the expert panel which was held in the form of Nominal Group Technique, the major sub-categories of Medical Education research priorities “35” were determined. The mentioned items were gathered after omitting and integrating similar cases and improving writing style of them.

In this phase, the number of extracted items was 35 which consisted of 12 items in teaching approaches, 7 items in educational management, 4 items in mentorship and educational guidance, 6 items in medical ethics and professionalism and 6 items in educational evaluation. After the extracted items were corrected and confirmed, they were developed into a questionnaire to be administered to the participants through 2 rounds of Delphi. The demographic information of the experts in first round of Delphi is shown in table 1.

**Table 1:** Demographic information of experts participating in first round of Delphi

**Variant**	**Number**	**Percentage**
**Occupational Status (Total number of participants: 33)**		
MD	7	21
Ph.D	26	79
**Job Experience in Years (Total number of participants:33)**		
Less than 10 years	12	36
11-20	9	28
21-30	5	15
More than 30 years	7	21
**Experience of teaching and activity in Medical Education in years (Total number of participants:33)**		
Less than 5 years	7	22
6-10	12	36
11-20	9	27
21-30	5	15
**Scientific Degree (Total number of participants:33)**		
Full professor	4	12
Associated professor	13	38
Assistant professor	16	48

**Table 2:** Mean±SD of the items fromthe experts’points of view afterfirst round of Delphi

**Row**	**Items**	**Mean±SD**	**Accept/Reject**
**Teaching approaches**			
**1**	Reform of education plans in medical education especially integration of basic sciences and clinical sciences in medicine	7.75±1.87	Accept
**2**	Designing teaching patterns based on lesson content in both theory and practice for medical students	6.43±1.66	Reject
**3**	Evaluation of the relationship between educational plans and educational goals of the field	7.48±2.07	Accept
**4**	Reform of content, implementation and evaluation of continuing medical education for faculty members	6.66±2.13	Reject
**5**	Assessment of CME program and workshops’ efficacy	7.15±2.21	Accept
**6**	Needs assessment of CME program (from medical students, General Physicians, and University professor)	7.94±2.63	Accept
**7**	Accreditation and standardization of CME program	7.69±2.66	Accept
**8**	Holding workshops for applying clinically educational approaches in students’ education	7.64±2.45	Accept
**9**	Holding team teaching workshops to educate professors	6.57±1.49	Reject
**10**	Development of electronic learning in university	7.36±1.65	Accept
**11**	Holding EBM workshops and extensive use of that in university	7.33±1.73	Accept
**12**	Holding communication skills workshops to have a proper relationship with patients	7.67±1.52	Accept
**Mean±SD of all fields: 7.92±1.35**			
**Educational management**			
**13**	Graduates’ roles and their properness with society needs	8.24±1.76	Accept
**14**	Strategic budgeting in education and assessment of economy role in medical education	7.45±1.32	Accept
**15**	Determining admission criteria for medical students, residents and the way of their optimization	7.45±1.66	Accept
**16**	Assessment of different factors affecting university (management, professors and students)	7.91±2.69	Accept
**17**	Role of science management and the way of its development in university	7.11±2.09	Accept
**18**	Assessment of policy making in medical education	7.41±1.32	Accept
**19**	Assessment of educational-functional management in university	6.72±1.34	Reject
**Mean±D of all fields: 7.48±1.58**			
**Mentorship and educational guidance**			
**20**	Assessment of mentor role in guiding and training students (higher education students and their theses)	7.67±1.92	Accept
**21**	Improvement in employingmore mentors in clinical fields	7.73±1.49	Accept
**22**	Designing proper mentorship pattern according to country status	7.33±1.56	Accept
**23**	Assessment of faculty members’ activities from spiritual aspects	6.55±1.52	Reject
**Mean±SD of all fields: 7.32±1.52**			
**Medical ethics and professionalism**			
**24**	Exploring teacher-student relationship and teaching medical ethics to students	8.16±1.72	Accept
**25**	Assessment of clients’ satisfaction about paying attention to medical ethics by professors	6.46±1.36	Reject
**26**	Professor education on professional ethics in medicine	8.34±1.05	Accept
**27**	Reducing medical errors by teaching medical ethics to students	7.76±1.14	Accept
**28**	Holding workshops for patient respect, designing and reforming patient rights protocol	8.19±1.82	Accept
**29**	Holding bad news workshop by professors according to spread of chronic diseases such as cancer	7.88±2.12	Accept
**Mean±SD of all fields: 7.63±1.26**			
**Educational evaluation**			
**30**	Applying new evaluation methods in evaluating residents in order to improve internal evaluation	7.41±2.18	Accept
**31**	Evaluating theoretical and practical exams and the ways of score analysis	7.31±1.63	Accept
**32**	Evaluation of existing teaching approaches and determining their role in educational progress and success in residency and board exams	7.52±2.25	Accept
**33**	Evaluation of educational and behavioral activities of professors	7.55±1.41	Accept
**34**	Reform of professors’ evaluation approaches and the way of their promotion regarding scholarship	7.25±1.43	Accept
**35**	Evaluation of medical students’ learnt issues in clinical environments	6.74±1.41	Reject
**Mean±SD of all fields: 7.28±1.52**			

As demonstrated in the table 2, Medical Ethics and Professionalism field with the mean±SD of 7.63±1.26 has gained the highest score and Educational Evaluation with the mean±SD of 7.28±1.52 has gained the lowest score among the major fields. “Professors’ Education about Medical Ethics” with the mean±SD 8.34±1.05 gained the highest score and another sub-category entitled “Assessment of clients’ satisfaction with professors and students’ paying attention to Medical Ethics” with the mean±SD of 6.46±1.36 gained the lowest score among all the items of **Medical Ethics and Professionalism. **After Delphi was done, according to previous studies, the items with scores less than 7 were omitted.

In two rounds of Delphi, the questionnaires with the accepted scores were resent to the participants of the second round to recheck the items’ status. Through this stage two items were added to the list due to their importance. These items were “Content reform, conducting and evaluation of CME program” and “Assessment of professors from the spiritual role aspect”.

Through second round of Delphi, the following items were excluded.

- “Editing teaching patterns proper to course content in clinical and practical lessons of medical students”,
- “Holding workshops to educate team-teaching approaches to professors” in Teaching Approaches field,
- “Assessment of functional-educational management at universities” in Educational Management field,
- “Assessment of clients’ satisfaction with paying attention to medical ethics by professors and medical students” in Medical Ethics and Professionalism field,
- “Evaluation of learnt issues by medical students in clinical wards of hospitals” in Educational Evaluation field.

## Discussion

Importance of research and its role in the development of a country is not unknown to anyone nowadays. So identifying research priorities in each field and barriers affecting them would be crucial. The present study was done to identify research priorities in Medical Education, their barriers and recommendations to remove the obstacles (16).

The present study is a qualitative one conducted by Delphi Method and NGT. The final items were extracted after conducting these mentioned phases (30 items in 5 major Medical Education field). Nemati et al. (2012) mentioned 6 fields of educational planning, educational evaluation, faculty members, educational and cultural management, CME program and gifted students and their findings are in line with the results of the present study (17).

Among major fields of this study, the field of Learning and Teaching strategies had the highest number of items which is due to the importance of issues mentioned in this field. In this field the item entitled “continuous needs assessment of educational program for medical students, General Physicians and university professors” gained the highest score and the item entitled “Holding team-teaching workshops for professors in teaching” gained the lowest score. Eventually, in this field 10 items were confirmed by experts. 

In the study conducted by Nemati et al. (2012) the participated professors considered educational planning and learning more important than the other fields and in this field, they emphasized educational planning in Medical Education and integration of various educational courses (17).

The next field was educational management in which 6 items were determined. “Graduates’ role and their proportionality with society needs” gained the highest score between other items; “Science management role and its development” gained the least score in this field. In this field, “Assessment of educational-functional management approach in university” was omitted after 3^rd^ round of Delphi based on experts’ opinions.

In Albert et al. study, researchers pointed to relationship between graduates’ role and their education with their professional career which is in line with the results of this study (18).

In the study conducted by Nemati et al. (2012), the item “graduates’ role and their professional duty and its proportionality with their education” gained the highest score which is in line with present study results. The item “Assessment of various cultural factors affecting university” gained the lowest score which is different from the results of the present study (17).

In mentorship and educational guidance 4 items were selected by experts  from which the item entitled “Assessment of professors’ function after spiritual role and competency criteria” gained the lowest score which was omitted in 2^nd^ round of Delphi. After third round of Delphi, the items were paid attention due to their importance. In this field, the item entitled “Improvement in employing more mentors (supervisors) in clinical environment” gained the highest score in this field. This could not been compared to other similar studies as it was the first time at universities to study this field with such items.

Clinical environment plays a major role and is a key factor in medical students’ learning because it provides a proper opportunity to face the clients and real problems. Therefore, clinical professors have a fundamental role in quality improvement of medical students’ learning in clinical fields. They should also have a proper behavior with students (19).

In Medical Ethics and Professionalism field, 5 items were extracted from which the item entitled “Reduction of medical errors by teaching medical ethics” gained the lowest score and the item entitled “Professors’ education on medical ethics” gained the highest score. Another item entitled “Assessment of clients’ satisfaction with medical ethics performance by professors and medical students” was omitted based on experts’ ideas. It is worth mentioning that this field gained the highest score among other ones. 

Professionalism is one of the most major issues in Medical Education. The importance of Medical Ethics and professionalism is one the necessary issues in Medical Education which must be considered in educational planning of this and other related fields. Recently, development of educational planning and fundamentals courses integration by emphasizing medical ethics and professionalism has become a basis for medical education in medical schools all over the world (20-21).

In a study conducted by Amini, the importance of Medical Ethics and Professionalism was found to be very important which is in line with the results of the present study (7).

The last field to assess was educational evaluation, in which 5 items were selected. The item entitled “Reform of faculty members’ evaluation and their promotion based on scholarship” gained the lowest score and the item entitled “Evaluation of educational and behavioral function of professors” gained the highest score. The item entitled “Evaluation of learned issues by medical graduates” was the item omitted by experts. This field gained the lowest score among the other ones.

It is also important to mention that the Evaluation field, especially Professor Evaluation, was also a major issue for research to Medical Education Researchers. Till now, there are several researches done under different issues in Professor Evaluation Field (22).

In recent years, there are several studies conducted at international universities under the goal of supporting patients, increasing clinical function effectiveness of physicians by the use of scholarship models (6).

## Conclusion

In previous years, several studies were conducted to determine research priorities but none of them determined detailed and functional research priorities in all fields. This makes our study different from the previous ones. There are some recommendations suggested by experts in order to make these research priorities more functional. 

One of the benefits of conducting the present study is that it is conducted by a qualitative method by which a short period of time is spent to gather data based on the experts and supervisors’ opinions. It also seems necessary to frequently determine research needs and priorities, and remove the obstacles existing in the way.  

**Recommendation:**

- Leading students theses and research proposals forward based on research priorities
- Holding national and international seminars and congresses based on determined priorities
